# A new age in structural S-layer biology: Experimental and *in silico* milestones

**DOI:** 10.1016/j.jbc.2025.110205

**Published:** 2025-05-08

**Authors:** Stephanie Grill-Walcher, Christina Schäffer

**Affiliations:** Department of Natural Sciences and Sustainable Resources, Institute of Biochemistry, NanoGlycobiology Research Group, University of Natural Resources and Life Sciences, Vienna, Austria

**Keywords:** cryo-electron microscopy, prokaryotes, protein structure, surface layers, X-ray crystallography

## Abstract

Surface (S-) layer proteins, considered as the most abundant proteins in nature, perform diverse and essential biological roles in many bacteria and most archaea. Their functions range from providing structural support, maintaining cell shape, and protecting against extreme environments to acting as a cell surface display matrix for biologically active molecules, such as S-layer protein-bound glycans, which facilitate interspecies interactions and cellular communication in both health and disease. The intricate, symmetric, nanometer-scale patterns of S-layer lattices have long fascinated structural biologists, yet only recent methodological advances have revealed detailed molecular insights. These advances include a deeper understanding of domain organization, cell wall–anchoring mechanisms, and how nascent proteins are incorporated into existing lattices. Significant progress in sample preparation and high-resolution imaging has led to the precise structural characterization of S-layers across various bacterial and archaeal species. Furthermore, the advent of deep learning–based structure prediction has enabled modeling of S-layer proteins in several largely uncultured microbial lineages. This review summarizes major achievements in S-layer protein structural research over the past 5 years, presenting them with a typical workflow for the experimental structure determination. For the first time, it also explores recent breakthroughs in computational S-layer modeling and offers an outlook on how *in silico* methods may further advance our understanding of S-layer protein architecture.

## Introduction to S-layers

**Figure 3:**
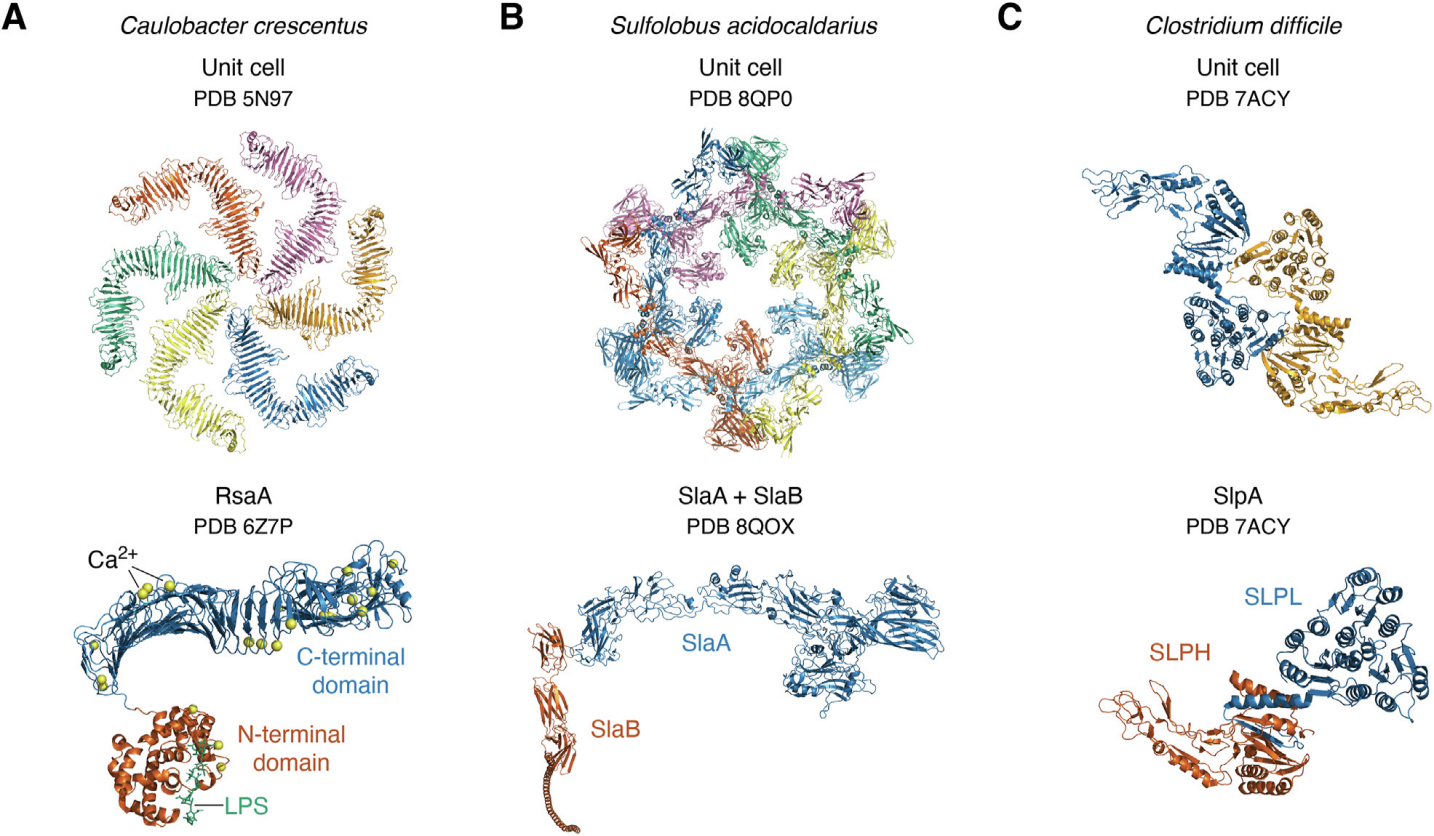
**Experimentally determined SLP structures.** Overview of three different experimentally determined structures. *A*, *top*: *Caulobacter crescentus* unit cell of hexameric RsaA (symmetry group p6) (PDB 5N97), *bottom*: single RsaA molecule (PDB 6Z7P) consisting of a C-terminal assembly domain (*blue*) and an N-terminal attachment domain (*orange*), where bound calcium ions are colored in *yellow* and bound LPS is colored in *green*. *B*, *top*: *Sulfolobus acidocaldarius* unit cell of hexameric SlaA (symmetry group p3) (PDB 8QP0), *bottom*: dimer of SlaA (*blue*, assembly protein) and SlaB (*orange*, attachment protein) (PDB 8QOX). *C*, *top*: *Clostridium difficile* unit cell of dimeric SlpA (symmetry group p2) (PDB 7ACY), *bottom*: One SlpA molecule (PDB 7ACY) consists of a low molecular weight subunit (SLPL, *blue*, assembly) and a high molecular weight subunit (SLPH, *orange*, attachment).

The outer surface of many prokaryotes, including bacteria and nearly all archaea, is adorned with a 2D, para-crystalline, proteinaceous layer known as cell surface (S-) layer (1, 2, 3). S-layer proteins (SLPs), which self-assemble into a closed lattice on the cell surface, are among the most abundantly synthesized and exported proteins in nature. An average-sized bacterium must produce approximately 400 copies of (often glycosylated) SLPs per second to fully coat its surface (2). SLPs typically have high molecular weights, are rich in hydrophobic amino acid residues, and frequently undergo *N*- or *O*-glycosylation—one of the most common posttranslational modifications (PTMs) in S-layers (4). Most SLPs studied thus far feature a modular domain architecture (5), with one domain driving lattice assembly (6) and another involved in anchoring the SLP to specific cell wall components. These anchors include peptidoglycan-bound secondary cell wall polymers (SCWPs) in Gram-positive bacteria (7), pseudomurein in archaea (2, 8, 9), lipopolysaccharides (LPS) in Gram-negative bacteria, and the mycomembrane in actinobacteria (10). In some S-layer systems, such as *Sulfolobus*, anchoring and assembly roles are split between two proteins (SlaA and SlaB, Fig. 3*B*) though most S-layers rely on a single SLP. While this dual-protein system is less energy-efficient, it offers greater adaptability by allowing surface-exposed proteins to evolve independently from the anchoring mechanism, which is preserved in the second protein (11).

Despite wide sequence diversity in SLP assembly domains, anchoring domains often share conserved motifs, such as the S-layer homology (SLH) domain comprised of ∼55 amino acid residues and commonly found in Gram-positive bacteria (5, 6, 12, 13, 14, 15, 16). Three SLH domains form a trimeric structure that binds noncovalently to SCWPs, as demonstrated by Sychantha *et al.* (17) and Legg *et al.* (18), through cocrystallization of SLH trimers from *Bacillus anthracis* and *Paenibacillus alvei* with synthetic analogs of the terminal sugar residues of SCWPs. Typically, conserved SLP residues face inward (toward the cell wall), while variable residues face outward, allowing adaptation to environmental changes and new interaction partners (19).

### Functional diversity and biological roles of S-layers

The considerable metabolic investment prokaryotes devote to maintaining S-layers underscores their critical role in survival and adaptation, particularly in extremophiles (20). While the full range of S-layer functions remains elusive, proposed roles span from providing biophysical and structural support to mediating cellular communication and pathogenicity, though direct experimental evidence remains limited (21, 22, 23, 24). A key structural role of S-layers is maintaining cell shape, especially in archaea, which lack complex cell wall architectures. S-layers also provide protection from environmental stressors such as high temperatures, radiation, desiccation, oxidation, and vacuum. This protective function is vital for extremophiles, for example*, Deinococcus radiodurans*, which relies on its S-layer to shield against ultraviolet-C radiation (25, 26), the halophilic archaeon *Haloferax volcanii*, which thrives in high-salt environments like the Dead Sea (27), and hyperthermophilic, acidophilic *Sulfolobus* species (11). Other organisms, like *B. anthracis* (28), the causative agent of anthrax, and *Clostridium difficile*, which affects the human colon (29), evade detection by the immune system by their S-layers, presenting a dynamic and complex cellular surface.

The pores, formed between SLP units during lattice assembly and, thus, occurring periodically in distinct shape, act as molecular sieves with remarkably precise exclusion limits (30). Buhlheller *et al.* recently proposed an evolutionary link between pore size and environmental adaptation. Species in harsh environments tend to have tighter lattices with smaller pores for increased protection, while others with more dynamic environments exhibit larger pores (31) to facilitate processes like nutrient uptake and enzyme secretion (*e.g*., sialidases scavenging sialic acid from host glycoproteins (32)) or withstanding changes in osmotic pressure, pH, or shear forces (33). In *D. radiodurans*, the S-layer pores can switch between, open and closed (plugged) conformations, though the physiological triggers for this transition remain unclear (34). Additionally, S-layers serve as a display matrix for biological signaling, displaying functional groups or covalently bound glycans that mediate intercellular interactions (9).

### S-Layer glycans: A potent but vastly unexplored mode of communication

The role of S-layer glycosylation is even more debated than the function of the S-layer itself. In electron microscopy or X-ray crystallography maps, glycans often appear as diffuse densities, typically inferred from known glycosylation sites *via* glycoproteomics (35). In cryo-EM studies, ambiguous densities are frequently attributed to glycans due to a lack of alternative explanations (36). The inherent flexibility of glycans further complicates precise structural determination. Gambelli *et al.* proposed that this flexibility promotes “glycan-guided S-layer assembly,” where restricted glycan movement incurs an entropic penalty, favoring interactions between the glycan-free protein domain interfaces, akin to cadherin assembly (37). Recent advancements in the neural network-based structure prediction tool AlphaFold 3, which now models glycans at defined sites, may offer new insights into this process (38).

From a biophysical perspective, glycans are thought to maintain a hydration layer around the cell that serves as a protective antifouling barrier (39) or aid in cellular adhesion (40). They may also stabilize the S-layer electrostatically, especially under acidic pH where deprotonation and hydrogen bond disruption are more likely (36, 41). Additional roles include the formation of a protective, mesh-like layer that impedes phage attacks (35) or partially occluding S-layer pores to block the entry of proteolytic enzymes (19, 11).

S-layer glycosylation is also linked to pathogenicity, with some pathogenic species displaying surface glycans that mimic host glycosylation to evade immune detection, for instance, the human oral pathogen *Tannerella forsythia* (42). Not only in *T. forsythia*, but also in *B. anthracis* (28), and *C. difficile*, (29), loss of the S-layer can impair both survival and virulence (28, 43, 44, 45, 46, 47, 48). Beyond pathogenicity, S-layer glycosylation has been shown to influence mating efficiency in the archaeon *H. volcanii* (49).

### S-layer protein assembly: Symmetry and energetic efficiency

In nature, large, regular structures often form *via* entropy-driven self-assembly to minimize free energy. S-layers exemplify this, as one or two dedicated proteins—typically just one—spontaneously assemble into 2D para-crystalline arrays (lattices) on prokaryotic cell surfaces, commonly facilitated by the presence of divalent cations like Ca^2+^ or Sr^2+^ (11, 27, 50, 51). Some S-layers, like the Sap protein from *B. anthracis* (28) and PS2 from *Corynebacterium glutamicum* (19), can also self-assemble *in vitro* without divalent ions, forming tubules or sheets.

A key challenge in structural studies of SLPs is their tendency to form 2D lattices rather than 3D crystals required for X-ray crystallography. To overcome this, methods have been developed to inhibit self-assembly, including nanobody binding or insertion of disruptive tags at key interface sites for assembly (28, 51, 52). Additional protocols allow controlled disassembly and reassembly of S-layers, either in solution or on solid supports, to facilitate structural studies (36, 39, 53, 54, 55). Disassembly is typically achieved using high-pH buffers (*e.g*., pH 10) or chaotropic agents, while reassembly occurs *via* dialysis at neutral pH in the presence of Ca^2+^ ions (11, 51, 56, 57, 58).

**Figure 1:**
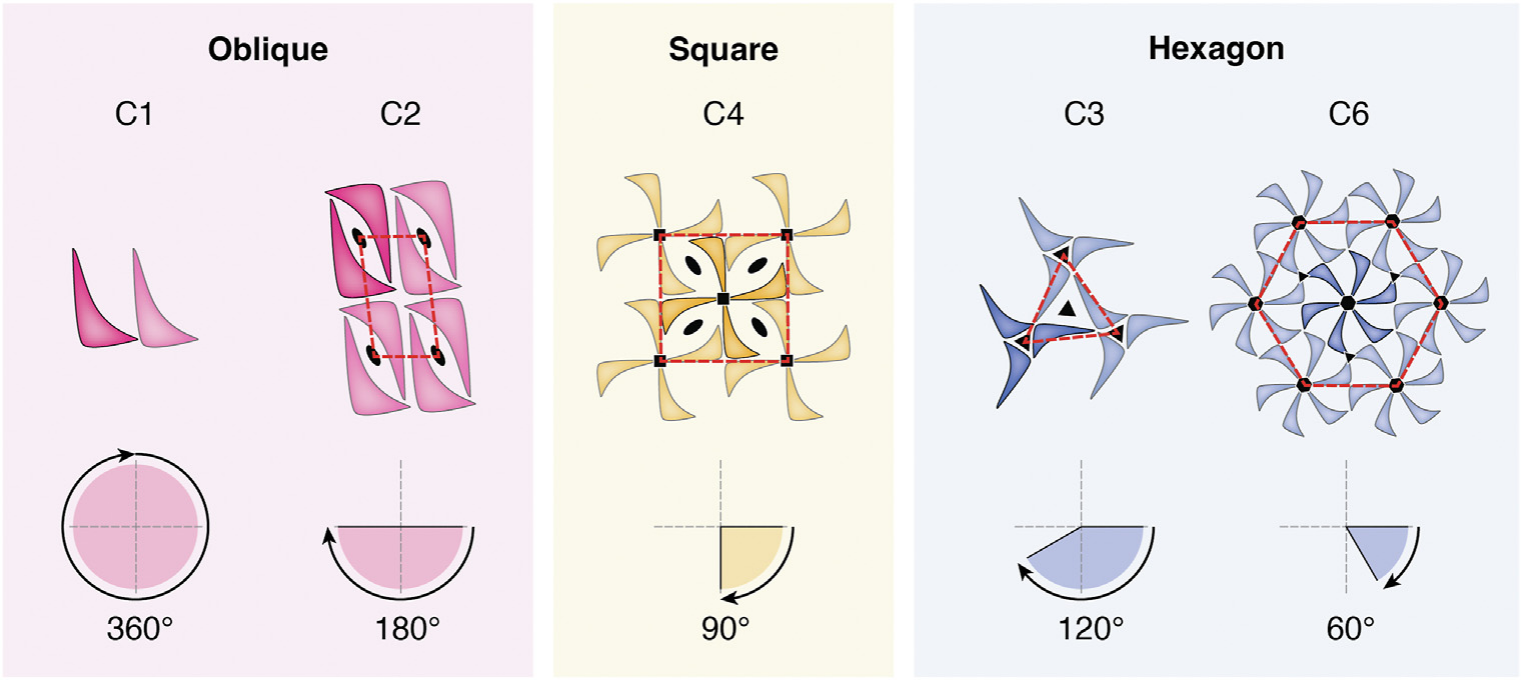
**Schematic illustration of known S-layer lattice types.** Rotational symmetries are displayed below each lattice type, with symmetry axes displayed by *black* symbols and outlined with *red* dotted outlines. Adjacent unit cells are rendered transparent for clarity.

**Figure 5:**
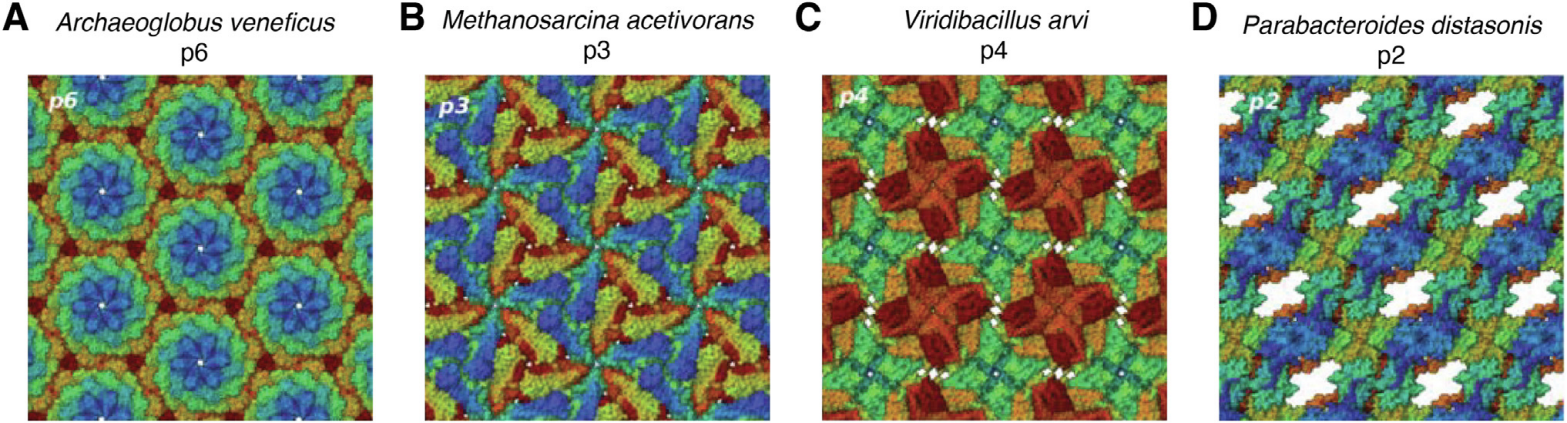
**Computationally derived SLP structures.***A*–*D*, overview of four different computationally derived S-layer structures and their corresponding space groups p6, p3, p4, and p2 (Taken from (63)).

The assembly domains of SLPs, visible in surface microscopy as the exposed parts of the S-layer lattice, create highly symmetric surface patterns adhering to basic principles of crystallography (1, 6, 59, 60). Among the 230 known crystal space groups (61), only five—p1, p2, p3, p4, and p6—are found in S-layer lattices (Fig. 1, examples shown in Figs. 3 and 5), reflecting the chirality of proteins, distinct “top” and “bottom” sides of S-layer planes, and the biological exclusion of mirror, inversion, or glide plane symmetries (62).

**Figure 2:**
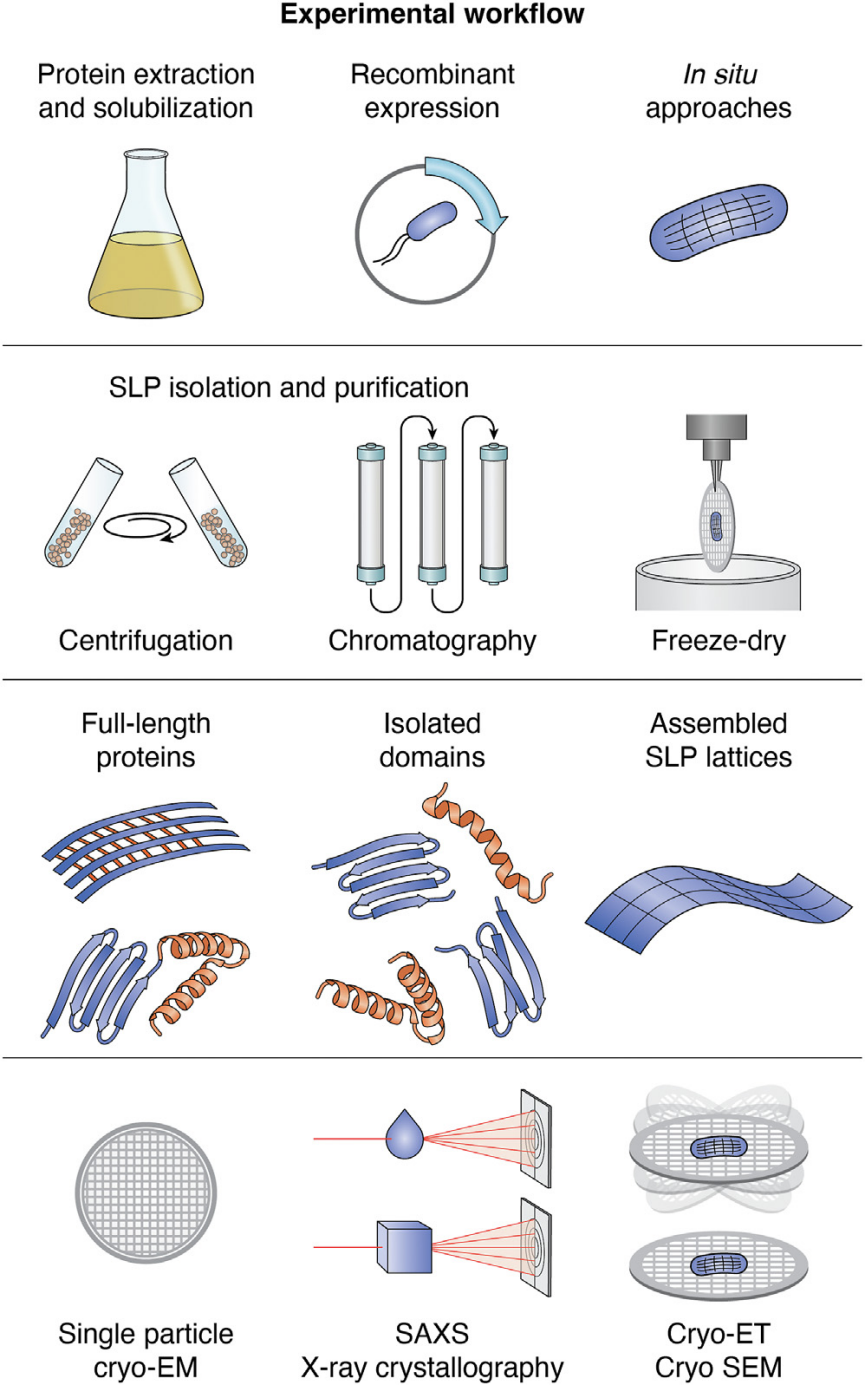
**General workflow for experimental SLP structure determination.** Experimental workflows typically begin with protein extraction and solubilization from the native organism using enzymatic digestion, detergents, or low-pH buffers. Alternatively, recombinant expression or *in situ* approaches may be employed. SLPs are then isolated by centrifugation and purified through successive chromatography steps, or cells are freeze-dried for direct *in situ* analysis. These methods yield full-length (including mutant) proteins, assembled SLP lattices, or isolated domains (truncated or tagged). Structural determination is subsequently performed using cryo-preservation techniques or nanobody-assisted X-ray crystallography.

**Figure 4:**
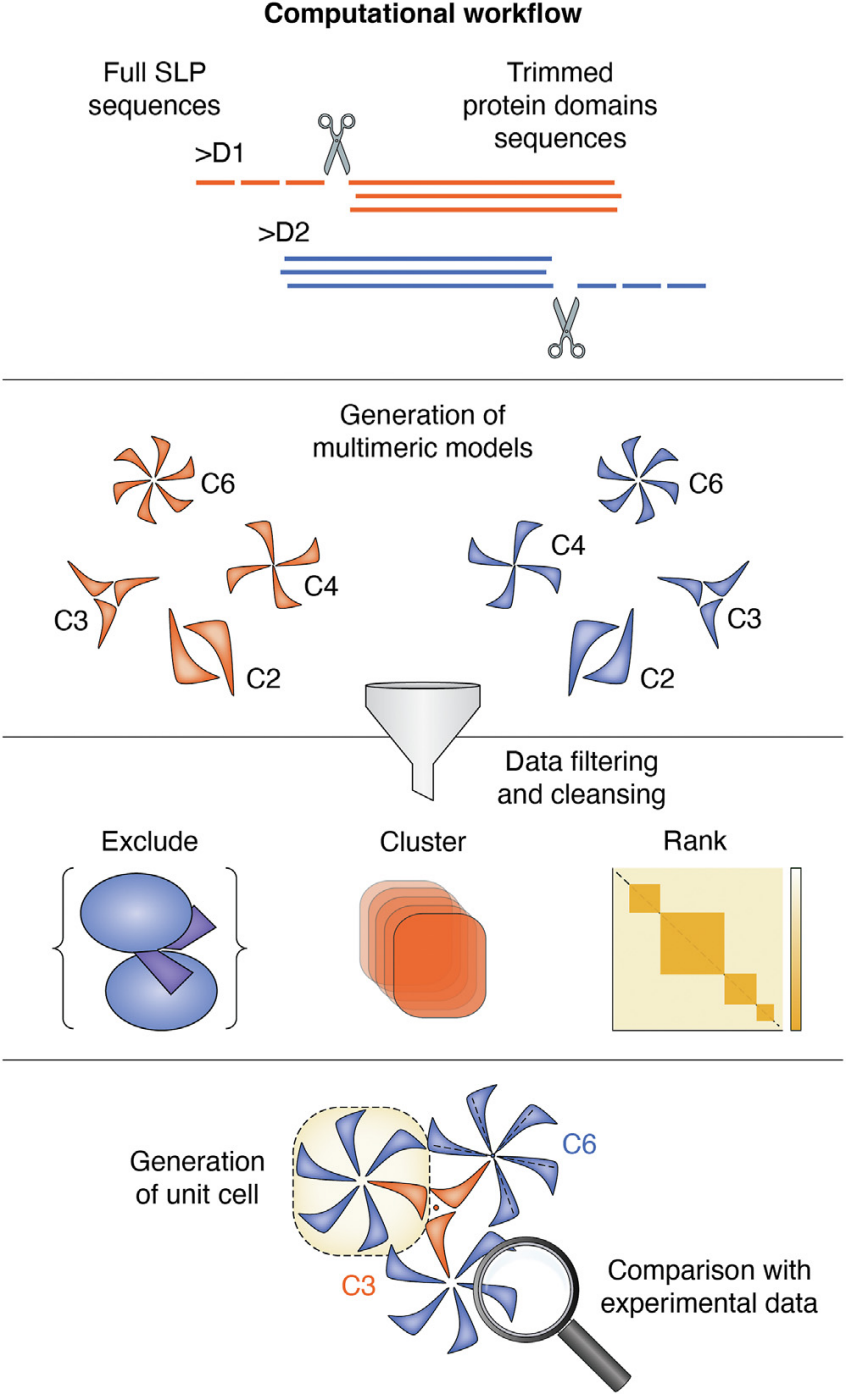
**General workflow for computational SLP structure determination.** Current computational workflows begin with full S-layer protein sequences or sequences of manually or automatically trimmed protein domains (excluding signal peptides or structurally irrelevant regions). Multimeric models are generated for each domain, followed by filtering, clustering, and ranking based on predefined quality parameters. Redundant or sterically clashing solutions are excluded. The most stable solutions for each domain are averaged, symmetrized, and superimposed to generate a final unit cell, which can then be compared to experimental data.

The striking patterns of S-layer lattices have intrigued structural biologists for decades. Today, single-molecule resolution has been achieved, revealing precise details of SLP domain arrangements, their attachment to the cell wall, and the role of divalent ions in lattice formation. Advances in sample preparation have been critical to these breakthroughs through addressing the challenges imposed by the self-assembling nature of SLPs (Fig. 2). Equally transformative are recent *in silico* tools, such as convolutional neural networks for protein structure prediction, which allow modeling of S-layer architectures from primary amino acid sequences alone (31, 63). These computational advances expand the study of SLPs beyond classical model organisms and facilitate the discovery of novel SLPs (63) (Fig. 4).

**Figure 6:**
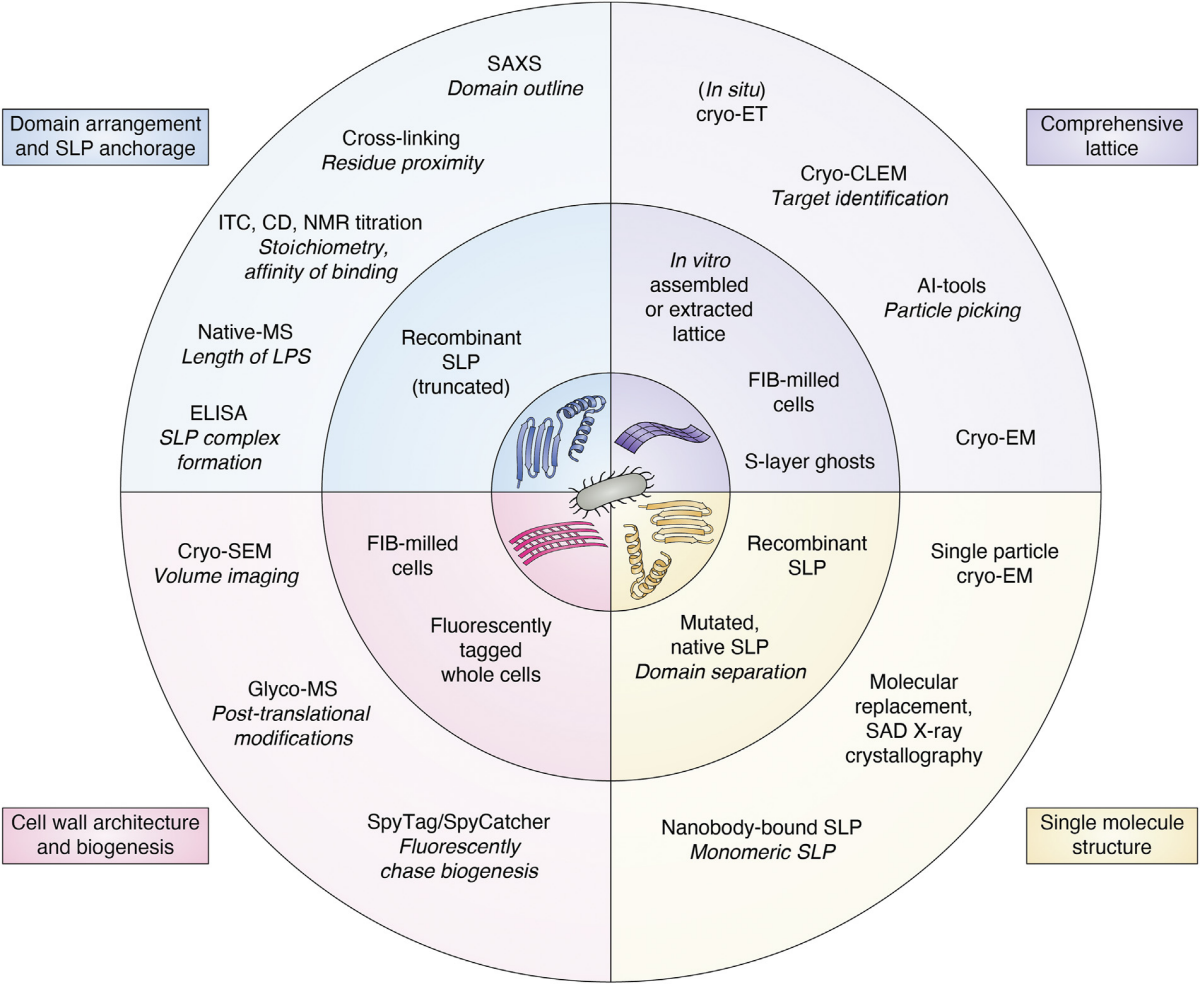
**Overview of techniques for SLP structure determination.** The overview is separated into four layers and four quarters: The outermost layer highlights the types of information obtained from structural studies. The next layer lists common visualization techniques. Below that, sample preparation methods associated with each technique are described. The innermost layer presents cartoon illustrations of the corresponding sample types.

This review highlights key milestones in SLP structure determination over the past 5 years, placing them within the broader context of the experimental workflow for structural analysis of these complex proteins. It also introduces, for the first time, recent *in silico* advancements in SLP structure determination and discusses potential future directions, illustrating how computational tools may deepen our understanding of SLP architecture. For a summary of structure analysis techniques, the types of samples they employ, and the insights they provide, see Figure 6.

## Experimental sample preparation strategies for S-layer characterization

Early SLP structural studies (3, 64, 65) focused on the surface-exposed regions of SLPs on whole cells to define unit cell geometry (such as center-to-center spacing, dimensions, and angles) and symmetry type (6, 33, 66). Today, advances in single-molecule techniques and cryogenic sample preparation provide much deeper insights into the molecular organization of S-layers and their (glyco)proteinaceous protein components (subunits). A general workflow is depicted in Figure 2.

Recent progress in SLP structural analysis falls into two main areas: (1) improved sample preparation, including optimized extraction protocols for native SLPs from diverse bacterial and archaeal sources, rational design of recombinant SLP truncations to dissect attachment and assembly domains, and advanced cryogenic *in situ* preparation; and (2) advancements in structure determination methods such as cryo-EM, nanobody-assisted X-ray crystallography, and small-angle X-ray scattering (SAXS), all benefitting from cryogenic workflows (Figs. 2, 6). Finally, developments in data analysis and automation of data collection have further advanced the field. A detailed discussion of these techniques lies beyond the scope of this review but can be found elsewhere (67, 68, 69, 70, 71, 72).

Sample preparation typically uses two sources: the native organism, either wild-type or genetically modified (*e.g*., with an enzymatic cleavage site between the SLP’s assembly and anchoring domains) (73), or recombinantly produced full-length or truncated versions of the SLP of interest, usually expressed in *Escherichia coli*. Mutation is limited to well-characterized, genetically tractable organisms, such as the Gram-negative bacterium *Caulobacter crescentus* (73, 74) or the Gram-positive *Geobacillus stearothermophilus* (51) (Figs. 2, 6). Sample preparation strategies depend on the study’s objective. Intact S-layer lattices are ideal for examining unit cell dimensions and arrangement on the cell surface but provide limited insight into molecular details such as anchoring mechanisms. In contrast, recombinantly expressed SLPs or their domains are commonly used to resolve single-molecule structures, yet they offer incomplete information on unit cell assembly. An overview of experimentally determined SLP structures is summarized in Table 1 and discussed in the following section.

**Table 1:** Recent advances in the structure determination of S-layer proteins in chronological order

Protein	Organism	Sample preparation	Visualization technique	Year	Reference
SbsC	*Geobacillus stearothermophilus,* G^+^	Purification from native organism, array formation with Ca^2^	Trehalose-embedded lipid monolayer crystals, electron microscopy, projection map of 7 Å	2007	(163)
SbsC, including N-ter SCWP-binding domain	*Geobacillus stearothermophilus,* G^+^	C-terminally truncated recombinant production	X-ray crystallography and molecular replacement with solved domains, 2.4 Å improved model of earlier publication including C-ter domains, SAXS for SLP assembly domain characterization, and interdomain arrangement CD to characterize binding of SbsC to SCWP	2003, 2008	(15, 164)
Sap-SLH domain	*Bacillus anthracis,* G^+^	Recombinant production of GST-tagged or His-tagged SLH-domain, synthesis of analogous terminal SCWP unit	X-ray (by SeMet-SAD), cocrystallization of Sap-SLH with analog of the terminal SCWP unit	2011, 2018	(16, 17)
C-ter repeat segment of MA0829	*Methanosarcina acetivorans,* archaeon	Recombinant production	X-ray (by SeMet-SAD) and inference of tandem structure by molecular replacement with the opposite domain	2012	(17)
Full-length SbsB	*Geobacillus stearothermophilus,* G^+^	Nanobodies raised against an assembly-incompetent, recombinantly produced SbsB with inserted HA tag, WT SbsB and rSbsB were both visualized	Nanobody-aided X-ray crystallography, nanobody-aided SAXS, cryo-EM, MD simulations, chemical cross-linking	2012	(51)
RsaA assembly domain	*Caulobacter crescentus,* G^-^	Extraction from native organism in low pH buffer and purification by ion exchange chromatography, reconstitution at physiological pH with Ca^2+^ or Sr^2+^, stalk shedding for improved image quality	X-ray crystallography combining S-SAD and molecular replacement, cocrystallization of assembly domain with Ca^2+^ and Sr^2+^, cryo-ET, and STA *in situ*	2017	(74)
Sap assembly domain	*Bacillus anthracis,* G^+^	Recombinant production of soluble Sap for incubation with camelid single-domain nanobodies	Nanobody-aided X-ray crystallography, nanobody-aided SAXS (confirming domain organization), cryo-EM on fully polymerized 2D purified Sap lattices and tubules	2019	(28)
SlaA and SlaB	*Sulfolobus,* archaeon	Isolation from native organism by incubation with sodium lauroylsarcosine at 37 °C, individual subunits were washed out, assembly in CaCl_2_	Negative staining (T)EM on full SLP SlaA–SlaB complexes and SlaB-depleted S-layers, cryo-ET, and STA *in situ*	2019	(11)
RsaA attachment domain	*Caulobacter crescentus,* G^-^	Isolation from native organism by low pH extraction and purification on ion-exchange chromatography and SEC, or mutant native organism carrying a TEV-cleavage site between NTD and CTD, on-column cleavage with His-TEV, acetic acid treatment of LPS	Single particle cryo-EM, cryo-ET with STA directly on cell stalks, MD simulations, and native MS for the binding of LPS-O-antigen to SLP NTD	2020	(73)
SDBC, main SDBC component: SlpA (DR_2577)	*Deinococcus radiodurans,* G^+^	Isolation of native complex by lysozyme treatment, homogenization, and centrifugation, solubilization in the nonionic detergent β-DDM for purification by ion-exchange chromatography and SEC	Negative staining EM on extracted and purified SDBC complexes. Follow up study: Single particle cryo-EM, cryo-EC on intact cells (for localization and orientation only)	2020–2022	(25, 84, 112)
Csg	*Haloferax volcanii,* archaeon	Extraction from native membranes with Concanavalin A	Cryo-EM on purified SLP sheets using a reference structure built by STA of cryo-ET on purified SLP with protein A gold	2021	(27)
SlpA	*Clostridium difficile,* G^+^	Isolation of native SLP by low pH extraction and centrifugation followed by SEC purification, recombinant expression of His-tagged SLP domains or domain variants, SLP ghosts created by enzymatic peptidoglycan digestion with endolysin	Cryo-EM on SL-ghosts or SLP fragments generated by mechanical disruption cryo-ET on S-layer ghosts on nanogold beads, X-ray crystallography combining S-SAD and molecular replacement on native and recombinant SLP	2022	(29)
SDBC	*Deinococcus radiodurans,* G^+^	cryo-FIB milling of whole cells stained with fluorescent membrane dye	(Super-resolution confocal microscopy for target identification), cryo-ET, cryo-CLEM, cryo-SEM volume imaging	2022	(113)
HPI protein	*Deinococcus radiodurans,* G^+^	Isolation of intermediate layer by dropwise addition of SDS and centrifugation FIB milling of whole cells	Single particle cryo-EM, cryo-ET of FIB-milled cells (to confirm that purified S-layers are the same as native ones), STA of cryo-ET data	2023	(26)
*Nm*SLP and ammonia-oxidizing machinery	*Nitrosopumilus maritimus,* archaeon	Purification of native cell envelopes by centrifugation in the presence of a detergent (CHAPS)	Single particle cryo-EM, cryo-ET with STA, MD simulations for ammonium-ion binding at the S-layer pores	2024	(35)
SlpA and SlpX (*L. acid.*), SplA (*L. amylo.*)	*Lactobacillus acidophilus* and *Lactobacillus amylovorus,* G^+^	Recombinant expression of three protein fragments per gene (*E .coli* NEB Lemo21 DE3 or *E. coli* B834 for SeMet), purification by affinity chromatography (Ni-NTA) and SEC	X-ray crystallography (Se and Hg-SAD or *ab initio* by ARCIMBOLDO_LITE or by molecular replacement), isothermal titration calorimetry for interaction between SlpA molecules, NMR for interaction of SlpA with cell wall	2024	(111)
PS2	*Corynebacterium glutamicum*, G^+^	Native S-layer genes re-introduced into reference strain, isolation of SLP in 1% SDS by homogenization and purification on 20% sucrose cushion *via* centrifugation	(Single-particle-) cryo-EM with C6 symmetry, refinement and model building using Alphafold 2 and ModelAngelo	2024	(19)
SlaA and SlaB	*Sulfolobus acidocaldarius,* archaeon	S-layer disassembly at basic pH and purification by SEC, exosome preparation for cryo-ET	Single particle cryo-EM (for SlaA) in combination with Alphafold 2 and cryo-ET with STA (for complete lattice with SlaB)	2024	(36)

β-DDM, n-dodecyl-β-D-maltoside; CD, circular dichroism; cryo-CLEM, cryo-correlative light and electron microscopy; cryo-EC, cryo-electron crystallography; cryo-EM, cryo electron microscopy; cryo-ET, cryo electron tomography; cryo-FIB, cryo focused beam ionization; cryo-SEM, cryo-scanning microscopy; N-ter, N-terminal; CTD, C-terminal domain; C-ter, C-terminal; LPS, lipopolysaccharide; MD, molecular dynamics; NTD, N-terminal domain; SAD, single wavelength anomalous diffraction; SAXS, small angle X-ray scattering; SCWP, secondary cell wall polymer; SDBC, S-layer deinoxanthin-binding complex; SDS, sodium dodecyl sulfate; SEC, size exclusion chromatography; SeMet, Seleno-methionine; SLH, S-layer homology domain; STA, Sub-tomogram averaging; TEM, transmission electron microscopy; WT, wild-type.

### Preparing fully assembled S-layer lattices for studies on unit cell dimensions

Beyond the SLP structure, the spatial arrangement of unit cells on the cell surface is also of interest. High-resolution *in situ* imaging of S-layers has been achieved using cryo-electron tomography (cryo-ET) or, for volumetric analysis, cryo-scanning electron microscopy (cryo-SEM). Reducing sample thickness significantly enhances image resolution, which can be accomplished, for example, by stalk shedding in *C. crescentus* (74) or generating “S-layer ghosts” through enzymatic peptidoglycan degradation with endolysin (29). Mechanical disruption of cells, such as homogenization with acid-washed glass beads, is another strategy to prepare thinner samples (29). In some cases, sample preparation inherently reduces cytosolic density, increasing transparency, as seen in *Sulfolobus* strains (11). Cryo-focused ion beam (cryo-FIB) milling, a more recent technique, enables precise thinning of samples *via* controlled ion beam ablation (75, 76, 77). When combined with nondestructive imaging methods, such as *in situ* cryo-SEM or cryo-TEM for high-resolution imaging of transferred ultrathin lamellae, cryo-FIB milling allows for 3D structural analysis. Originally developed for *E. coli* in the early 2000s (78) (reviewed in (79)), this approach has been significantly refined through advancements in cryo-preparation, imaging, and data analysis. For further insights on improving cryo-EM and cryo-ET resolution, see Ochner and Bharat (67).

### Single-molecule approaches for SLP analyses

Single-molecule studies are key to resolving the detailed structure of SLPs. While they often allow extrapolation of unit cell organization based on known symmetry and dimensions, they rarely address the global S-layer lattice arrangement. Such studies have been performed on native (sometimes mutated) SLPs extracted from organisms like *C. crescentus* (73, 74) and on recombinantly expressed SLPs or their domains. Native extraction preserves proteins in their most physiological state, including PTMs. A common method for isolating SLPs from both Gram-positive and Gram-negative bacteria involves low-pH buffer extraction (29, 73, 74, 80). Alternatively, strong chaotropic agents, such as 5 M lithium chloride, 5 M guanidine hydrochloride, or 6 M urea, are frequently used (81).

For S-layers anchored primarily *via* hydrophobic interactions, such as in *C. glutamicum* (19, 82) and the extremophile *D. radiodurans,* extraction with 10% (w/v) SDS is effective and enabled isolation of the hexagonally packed intermediate (HPI) layer *in D. radiodurans* (26, 34, 83). Alternatively, lysozyme treatment followed by solubilization in n-dodecyl-β-D-maltoside and has been used to extract the native deinoxanthin-binding S-layer complex (SDBC) in *D. radiodurans* (25, 84). This milder approach preserves both hydrophilic and hydrophobic components, including minor subunits. In archaea, ultracentrifugation often suffices to separate the S-layer from cellular components, but protocols using sodium lauroylsarcosine—a surfactant commonly used in membrane protein solubilization—at 37 °C, followed by pellet purification, have also been applied to extract SLPs like SlaA and SlaB from *Sulfolobus* species. A subsequent washing step of the S-layer with a buffer containing low concentrations of divalent cations and SDS at 37 °C facilitates subunit dissociation (11).

SLP purification for single-molecule studies typically involves ion exchange chromatography, as shown for the SLP RsaA from Gram-negative *C. crescentus* (73, 74) and SlpA, the SLP from Gram-positive *C. difficile* (Fig. 3*C*) (29, 80), followed by size-exclusion chromatography for final purification (29, 73, 74, 84). Recombinant SLPs are often purified *via* affinity chromatography using polyhistidine (His) tags. Additionally, the archaeal SLP Csg from *H. volcanii* has been successfully purified using a Concanavalin A column, which selectively binds the mannose-rich glycans of this SLP (27).

When SLPs contain repetitive motifs, expressing a single domain recombinantly can suffice. For instance, in *Methanosarcina acetivorans*, whose SLP forms a dimer of two tandem repeats, only the C-terminal repeat was expressed in *E. coli* (Rosetta strain) for crystallization (computationally derived structure shown in Fig. 5*B*). High sequence similarity between the C- and N-terminal domains, especially at the dimer interface, allowed homology modeling to infer the full tandem structure, bypassing the need to study the entire SLP dimer (85).

### Investigating domain arrangement and cell wall–anchoring mechanisms

With a resolved SLP structure, researchers can investigate the specific mechanisms of cell attachment and domain organization, unveiling a possible hypothesis on S-layer assembly. Detailed analysis of domain architecture and anchoring often relies on recombinantly expressed SLP segments. Compared to extraction from the bacterial source, recombinant production offers greater flexibility, enabling the addition of purification tags to boost yield and purity, the insertion of assembly-inhibiting tags to prevent lattice formation, and targeted point mutations. The following examples illustrate how recombinant approaches facilitate precise studies of SLP domain organization and interactions. However, these findings typically require validation against native S-layers to ensure biological relevance, such as comparing cryo-EM data from recombinant proteins to *in situ* cryo-ET maps. Truncated recombinant SLPs or their domains are usually expressed in *E. coli* strains (*e.g*., BL21, Rosetta) and purified *via* affinity- and size-exclusion chromatography. Some SLPs, such as SlpA from *C. difficile*, tend to form inclusion bodies; SlpA is naturally cleaved into high- and low-molecular-weight subunits that co-assemble into the S-layer. Lanzoni-Mangutchi *et al.* used ELISA assays on recombinant SlpA subunits bearing point mutations to assess complex formation and stability, identifying key residues essential for subunit interaction through antibody-based detection and horseradish peroxidase readout (29). Mutations at critical sites disrupted subunit interactions.

In another study, Baranova *et al.* employed photoactivatable, biotin-tagged hetero-bifunctional crosslinkers to map proximity relationships between a surface-exposed cysteine engineered into domain II of SbsB and neighboring peptides within 22 Å distance of the assembled SbsB lattice (51). SbsB contains three N-terminal SLH motifs for membrane anchoring, followed by six domains (II-VII). Defining domain positioning is often complicated by structural flexibility, as seen in the elongated structures of SbsC from *G. stearothermophilus*, Sap from *B. anthracis* (28), or the triangular prism structure of SlpA from *C. difficile* (Fig. 3*C*) (29). This crosslinking approach precisely located domain II within the proposed S-layer structure. To investigate the cell wall–anchoring mechanism of the S-layer protein SpaA of *P. alvei*, a recombinant truncation of the SLP, comprised of soluble SHL-domain trimer with synthetic fragments of the bacterium’s SCWP, was performed. Two mutually exclusive binding grooves in the trimer were identified, with the terminal, pyruvylated *N*-acetylmannosamine residue identified as the crucial cell wall ligand (18, 86). A comparable S-layer–anchoring mechanism was also determined in *B. anthracis* (87).

## Innovations in S-layer structure imaging techniques

Visualization of carefully prepared samples is central to S-layer structural studies. While negative staining EM of extracted and polymerized SLP complexes remains valuable for rapid *ab initio* structural insights and as a reference for more advanced techniques (*e.g.*, 11, 19, 28, 29, 84), the past decade has seen major advances with the rise of cryo-EM and cryo-ET. These techniques are often combined with subtomogram averaging (STA), which improves resolution and reduces noise by aligning and averaging 3D volumes, analogous to 2D single-particle reconstruction (88). Previously, researchers relied on crystallizing SLP subunits or studying intact lattices. Today, cryo-EM and cryo-ET allow structural analysis of large, purified protein complexes at single-molecule resolution, frozen in near-native states.

Despite growing enthusiasm for cryogenic methods, X-ray crystallography has also advanced and still offers superior resolution than cryo-EM and cryo-ET (89, 90). However, SLP self-assembly into 2D lattices limits 3D crystallization, historically hindering its use. Recent innovations, including nanobody-mediated assembly inhibition, steric tag insertion, and mutation of key assembly residues, have helped to overcome these limitations. Two key techniques widely adopted in recent S-layer X-ray crystallography studies to address the ‘phase problem' are molecular replacement and single anomalous dispersion (SAD) phasing. The phase problem refers to the lack of phase information necessary for electron density map calculation from diffraction data. Molecular replacement addresses this by using known structures—such as homologs, domains of interest, or more recently, computationally predicted models—to estimate phases (52). For instance, Oeffner *et al.* (91) proposed a workflow combining AlphaFold-predicted models (to generate an overall structure and identify regions of uncertainty) with cryo-EM or X-ray data (to resolve uncertainties regarding domain placement and flexible loops), refining structures in ISOLDE, a physically realistic environment structural refinement (92). SAD, in contrast, is effective when no prior structural information is available. It exploits anomalous scattering from marker atoms, such as native sulfur (S-SAD), selenomethionine-substituted methionine (SeMet-SAD), or heavy atoms, to calculate phase information directly from the crystal (93). SeMet X-ray crystallography involves the recombinant expression of proteins in auxotrophic bacterial strains (*e.g*., *E. coli* strain B834 (DE3)) or yeast strains, with methionine substituted by SeMet in the growth medium. SeMet typically provides stronger X-ray scattering than native sulfur, thus offering superior phasing power, although the incorporation efficiency of SeMet can vary, and low expression yields of labeled proteins remain a challenge.

Further technical details are available in specialized reviews (94, 95).

Elucidating quaternary structure and understanding protein complex formation is central to S-layer structural biology, but challenging with X-ray crystallography alone, as biological interfaces are often hard to distinguish from crystal-packing artifacts. Estimates suggest that over 10% of Protein Data Bank (PDB) assemblies may be nonphysiological (96, 97). Consequently, X-ray data are typically complemented by other methods. Efforts, particularly in the field of deep learning, are ongoing to better differentiate physiological from nonbiological interfaces in X-ray crystallography (98).

### Structure determination of single-molecule SLPs

One of the most influential studies in recent S-layer structural biology is the work by von Kügelgen *et al.* on the Gram-negative bacterium *C. crescentus* and its SLP, named RsaA (73) (Fig. 3*A*). This study is notable for overcoming the challenges of studying Gram-negative SLPs, which are difficult to isolate due to their attachment to LPS, and for being among the first to combine multiple structural techniques to resolve the complete S-layer architecture and its attachment mechanism to LPS at molecular detail. Despite *C. crescentus* being a well-studied model organism, this work is one of the first to achieve a full S-layer model for a Gram-negative organism.

Building on prior data from the C-terminal assembly domain of RsaA—previously resolved *via* X-ray crystallography using holmium SAD and molecular replacement (74)—the study provided novel insights into the N-terminal attachment domain, which had previously failed to crystallize. The researchers proposed a functional separation between the “lattice-forming” and “membrane-anchoring” domains of RsaA (blue and orange domains in Fig. 3*A*). They engineered a TEV cleavage site into native RsaA to separate the N-terminal domain, which was then complexed with acid-treated, natively purified LPS (containing only the core oligosaccharide and O-antigen, devoid of lipid A) and analyzed using single-particle cryo-EM. The study culminated in an *in situ* validation of the S-layer model on *C. crescentus* cell stalks *via* cryo-ET with STA, achieving near-atomic resolution.

The group later expanded their research to detailed structural studies of SLPs from the haloarchaeal model organism *H. volcanii* (27) and the hyperstable, highly interconnected but porous S-layer of the Gram-positive bacterium *D. radiodurans* (26, 99). They proposed that a single SLP—the “HPI-layer surface protein”—constitutes the entire *D. radiodurans* S-layer, based on prior biochemical (100), genetic (101), and topographic studies (34, 102, 103, 104, 105, 106, 107). These studies demonstrated the extreme stability of isolated HPI protein under harsh conditions (*e.g*., detergent at high temperatures) and identified SlpA as a tether between the outer membrane and the peptidoglycan, not a structural S-layer component (99). The HPI protein comprises seven consecutive Ig-like domains, with the five C-terminal domains forming the S-layer lattice and the two N-terminal domains likely mediating membrane anchoring, a common functional division in SLPs (5). No additional protein densities were detected in the cryo-EM maps.

More recently, von Kügelgen *et al.* resolved the S-layer of the ammonia-oxidizing archaeon *Nitrosopumilus maritimus* (35), which is structurally similar to the Csg protein in *H. volcanii.* Combining single-particle cryo-EMs and cryo-ET-STA, they visualized the hexagonally symmetric (p6) lattice using sonicated native cell envelopes and protein A gold labeling for cryo-ET. Structural analysis revealed pentameric defects in the p6 lattice, contributing to S-layer integrity and continuity, similar to findings in *H. volcanii*. Such defects commonly occur near areas of high lattice curvature (*e.g*., bacterial cell poles) or regions affected by external stress or new SLP incorporation, where perfect crystalline order is difficult to maintain.

Although X-ray crystallography remains challenging for SLP structure determination, some recent successes have emerged. A notable example is the tightly packed S-layer of *C. difficile*, composed of SlpA, which undergoes posttranslational cleavage into a high- and low-molecular-weight subunit (blue and orange subunits in Fig. 3*C*) (29). The complete structure of this SlpA complex was resolved using S-SAD X-ray crystallography of recombinantly expressed SlpA subunits, combined with molecular replacement of individual interacting domains. To confirm that the crystal structure accurately reflected the native 2D *C. difficile* S-layer lattice, the study employed cryo-EM on mechanically fragmented cell surfaces and cryo-ET experiments on S-layer ghosts (enzymatically digested peptidoglycan preparations immobilized on nanogold beads). These experiments revealed that the lattice, exhibiting native-like parameters, could still form even when nearly half of the low-molecular-weight SlpA subunit was removed, suggesting that lattice formation is primarily driven by the high-molecular-weight subunit.

### Nanobody-aided SAXS and nanobody-aided X-ray crystallography for enhanced resolution

Nanobodies have been instrumental in preventing SLP self-assembly, enabling monomer crystal formation and facilitating SAXS and X-ray crystallography studies. The first application of nanobody-assisted crystallography in S-layer research was conducted by Baranova *et al.* (51), who investigated the structure of SbsB from the Gram-positive bacterium *G. stearothermophilus*, a seven-domain SLP with three N-terminal SLH motifs for anchoring and a C-terminal assembly domain. Nanobodies were raised against an assembly-incompetent SbsB mutant (engineered with a hemagglutinin tag at a site previously identified to inhibit assembly (108)), allowing successful crystallization of both mutant and WT SbsB. X-ray crystallography, supported by heavy atom tracking, resolved the structure at a resolution of 2.4 Å. The study also employed nanobody-aided SAXS experiments (relying heavily on protein solubility) for *ab initio* shape reconstruction, along with cryo-EM of *in vitro* (calcium-dependent) assembled SbsB lattices to resolve the continuous p1 lattice. Additionally, photoactivatable crosslinking and MD simulations aided domain arrangement refinement.

Fioravanti *et al.* (28) applied a similar strategy for structure determination of the SLP Sap from *B. anthracis*, a species with two growth phase–dependent SLPs-Sap (exponential phase) and extractable antigen 1 (EA1, stationary phase) (109). Both SLPs contain N-terminal SLH-anchoring domains and C-terminal p1-type lattice forming regions, stabilized by calcium. Sap and EA1 share a “beads-on-a-string” multidomain architecture, common in SLPs from *G. stearothermophilus*, *H. volcanii* (27), and *D. radiodurans* (26) despite low primary sequence similarity (5). Recombinant Sap was crystallized using two nanobodies that blocked assembly, yielding a 2.7-Å structure consistent with prior negative-stain EM data (110). Nanobody-aided SAXS further validated the domain organization. Fluorescently labeled nanobodies also disrupted native S-layers *in vivo*, as shown by SEM, TEM, fluorescence, and light microscopy. In a subsequent study, the group resolved the EA1 structure at 1.8 Å (52). To overcome EA1’s strong self-assembly tendency, it was purified under denaturing conditions (*i*.*e*., 8 M urea), and a nanobody screen identified two nanobodies that enabled 3D crystallization. Structure determination used X-ray crystallography, combining molecular replacement with AlphaFold 2–predicted EA1 domains and nanobody structures. As with Sap, cryo-EM on an *in vitro*–assembled EA1 lattice reconstructed the overall S-layer lattice.

### Mapping domain arrangement and SLP attachment to the cell wall

Direct visualization of SLP domain organization is rare and often relies on indirect methods, which are based on data fed into computational models to guide *in silico* structure generation. Similarly, SLP attachment to specific cell surface moieties is typically characterized through biochemical assays rather than direct observation. Accurate determination of both domain arrangement and SLP attachment mechanisms usually requires pure, natively isolated or recombinantly expressed SLPs (or functional fragments). Techniques such as chemical crosslinking and SAXS are widely used to map proximal residues and infer domain organization, as shown in studies on SbsB from *G. stearohermophilus* (51) or Sap from *B. anthracis* (28).

The SLP SbsC from *G. stearothermophilus* was recombinantly produced to study its interaction with SCWP extracted and purified from native sources. Binding affinity and stoichiometry were determined using isothermal titration calorimetry, while circular dichroism (CD) revealed conformational changes in the protein upon SCWP binding (15). In a more recent study, the same group (111) focused on two pathologically relevant *Lactobacillus* strains, *Lactobacillus acidophilus* and *Lactobacillus amylovorus*, expressing functional fragments of the SLPs SlpA and SlpX, which form a p2-symmetric S-layer—either independently (SlpA in *L. amylovorus*) or together (SlpA and SlpX in *L. acidophilus*, with SlpA as the dominant SLP). These fragments were crystallized for in-depth structural analysis by X-ray crystallography and investigated in functional studies using isothermal titration calorimetry and NMR titration. The results provided new insights into the role of SlpX in the p2 S-layer lattice.

In the aforementioned study by von Kügelgen *et al.* on the N-terminal domain of RsaA from *C. crescentus* (Fig. 3*A*), the domain structure was resolved and its attachment mechanism to the outer membrane was revealed (73). Cryo-EM analysis of the RsaA–LPS complex identified specific N-terminal RsaA residues binding the LPS O-antigen, with Ca^2+^ ions essential for attachment and stabilization of the S-layer. Chelation by EGTA led to disassembly, which was reversed upon Ca^2+^ reintroduction. To further refine the understanding of the SLP–LPS interaction, native mass spectrometry was used to assess LPS chain length, while MD simulations provided detailed insights into the RsaA–LPS interface and Ca^2+^-mediated stabilization.

In a follow-up study, Herdman *et al*. explored the role of Ca^2+^ using fluorescently labeled RsaA and all-atom MD simulations (50). The team experimentally confirmed 22 predicted Ca^2+^-binding sites on RsaA. MD simulations showed most sites stably bound to Ca^2+^, though two exhibited enhanced stability upon binding to Mg^2+^ instead. These findings were validated by cryo-EM and long-wavelength X-ray diffraction, with Holmium ions substituting for Ca^2+^ ions *in vitro*, which allows for cryo-EM comparisons between Holmium- and Ca^2+^-bound RsaA structures (73). Long-wavelength X-ray anomalous diffraction on RsaA’s C-terminal crystals further confirmed Ca^2+^ positions. The results established that Ca^2+^ is critical not only for S-layer attachment to LPS but also for forming a closed, continuous, predominantly p6 lattice essential for cell surface integrity and bacterial growth. Importantly, Mg^2+^ could not fully substitute for Ca^2+^, highlighting its unique role in S-layer assembly.

### Integrating single molecule structures into comprehensive lattices

As highlighted throughout this review, single-molecule cryo-EM and X-ray crystallography often complement cryo-ET analyses of native cells or assembled SLP lattices, together providing a detailed and comprehensive view of S-layer architecture (*e.g*., in *D. radiodurans* (26) and *H. volcanii* (27)) as recently reviewed in (71, 89)).

Two independent research groups, Farci *et al.* (25, 84, 112) and Sexton *et. al.* (113), employed cryo-EM and cryo-ET to explore the S-layer in *D. radiodurans.* Both groups presented evidence for a multiprotein complex decorating the cell surface of *D. radiodurans*, contradicting von Kügelgen’s suggestion of a single, long HPI surface protein (26, 99). One such complex, SDBC, involves the protein SlpA (DR_2577), identified by von Kügelgen *et al.* as the “membrane tether” (99) and involved in forming the interpore regions of the S-layer. Farci *et al.* highlighted SlpA’s striking sequence similarity to known porins, suggesting that it functions as a porin in addition to its structural role in the S-layer. The SDBC is implicated in the organism’s resistance to UV radiation and desiccation (114, 115). Farci *et al.* refined the protocol for isolation and purification of the native S-layer of *D. radiodurans*, enabling the identification of all potential subunits through mass spectrometry (116). Cryo-EM revealed a large (∼800 kDa) triangular particle, identified as the polyproteinaceous SDBC, the largest known porin complex at the time. The complex exhibited no imposed symmetry and featured three key regions: a large β-barrel “donut-shaped” structure integrating into the outer membrane, a stalk region possibly extending to the inner membrane, and a collar region between the outer membrane and periplasm (25, 112). Cryo-electron crystallography and cryo-ET projections of cell wall fragments showed six triangular pore complexes, each with a p3 symmetry, surrounding a larger pore, forming the characteristic p6 lattice. The SDBC was characterized for its cofactor binding and transport properties, linking its antioxidant and ultraviolet-C quenching functions to its structure (115). Sexton *et al.* also confirmed SDBC's association with the cell envelope but focused more on the inner membrane and subcellular features rather than the surface-exposed portion of the S-layer (113). These contrasting studies underscore the ongoing refinement of the S-layer model in *D. radiodurans*, fueled by advances in cryo-EM and cryo-ET techniques.

Cryo-ET, independent of cryo-EM, has also been successfully used to reconstruct complete S-layer models. For instance, Gambelli *et al.* used *in situ* cryo-ET combined with STA to confirm and extend earlier negative-staining EM data on the p3 S-layer of *Sulfolobus* species (11). The S-layer in *Sulfolobus* consists of two heavily glycosylated SLPs, SlaA and SlaB (Fig. 3*B*). SlaA is essential for *in vitro* lattice formation, driving assembly and determining geometry, while SlaB contributes to lattice stability and is proposed to anchor the S-layer to the membrane *via* an N-terminal transmembrane helix. The study showed that SlaB could be removed from the S-layer by washing with *N*-laurylsarcosine, enabling a comparison of the fully assembled and SlaB-depleted S-layer. Using difference maps derived from cryo-ET-STA data, the researchers inferred the positioning and assembly of the S-layer, creating a comprehensive structural model based solely on cryo-ET (11).

### Cryo-ET correlated workflows for 3D S-layer imaging for volumetric visualization

Although cryo-ET is limited to visualizing a sample’s surface, it can be combined with other techniques to create layer-by-layer volumetric image stacks. A challenge is identifying macromolecular targets within the crowded environment of cellular surfaces, which often leads to a poor signal-to-noise ratio and lower resolution.

To overcome this, (cryo-) fluorescence light microscopy continues to play a crucial complementary role. By tagging fixed, and therefore relatively static, cellular proteins with fluorophores or by using immunolabeling, precise localization can be achieved, enabling correlative light and electron microscopy. In a notable example, Sexton *et. al.* showcased a robust, user-friendly workflow for imaging *D. radiodurans* cell walls (113). This workflow is especially useful given the challenges of imaging large cells (∼4 μm in diameter, such as those from *D. radiodurans*) *via* cryo-ET. The team employed continuous FIB milling to selectively target specific cellular locations, followed by high-resolution cryo-ET imaging. Serial milling and cryo-SEM were used to generate volumetric stacks, providing context for the cryo-ET data within the overall cell wall architecture. Cryo-ET workflows have also become more autonomous through deep-learning integration, improving the critical particle-picking stage.

For instance, Rice *et al.* developed TomoTwin, a user-friendly, open-source deep metric learning algorithm (available at github.com/MPI-Dortmund/tomotwin-cryoet) that simplifies particle identification. Users only need to provide a representative molecule for each protein of interest, and the tool autonomously locates similar particles across a cryo-ET dataset. Alternatively, Moebel *et al.* introduced DeepFinder, a semi-automated convolutional neural network designed for fast particle identification. Available as a Napari plugin and fully open-source at github.com/deep-finder, DeepFinder supports the analysis of multiple molecule species simultaneously and requires minimal user input, improving speed and accuracy compared to conventional methods.

Another notable tool is DeePiCt (Deep Picker in Context), which specializes in particle detection within cellular contexts, even for low-abundance or low-density molecules (available at github.com/ZauggGroup/DeePiCt) (70). Additionally, Powell and Davis (117) adapted Deep Reconstructing Generative Networks (cryoDRGN), a tool originally developed for single-particle cryo-EM, into tomoDRGN, designed for cryo-ET, to capture both compositional and conformational heterogeneity in cryo-ET particles (available at github.com/bpowell122/tomodrgn), making it particularly valuable for studying dynamic molecules that undergo continuous conformational changes.

Numerous other open-source tools are available, each with unique capabilities. Researchers are encouraged to explore these options and stay updated on development to enhance cryo-ET analysis.

### Mechanistic visualization of S-layer biogenesis

The combined application of fluorescence microscopy and cryo-ET has significantly advanced our understanding of S-layer formation, its relationship to cell growth, and the spatial dynamics of SLP insertion.

Recent studies on *C. crescentus* (50, 118) and *C. glutamicum* (19) used the SpyTag/SpyCatcher system to fluorescently trace and localize newly synthetized, mutated, and Spy-tagged SLPs. These SLPs were labeled with covalently binding, fluorescent catcher tags (catcher-mCherry or catcher-GFP), enabling the differentiation between “old” and nascent SLPs during exponential growth. Sogues *et al*. employed fluorescence microscopy, while Herdman *et al.* combined this approach with cryo-correlative light and electron microscopy. In the latter, sites of new SLP insertion were localized using cryo-fluorescence microscopy and correlated with cryo-ET images. Both studies revealed that nascent SLPs are predominantly incorporated at the cell poles of *C. glutamicum* and *C. crescentus*. Higher-resolution cryo-ET further revealed structural disruptions in the S-layer at these insertion sites, such as overlaps, missing rows of hexamers or line defects where two S-layer sheets converge. Herdman *et al.* also identified the presence of a pool of unassembled SLPs within the periplasmic space between the outer membrane and the existing S-layer, an observation that has already been discussed more than 30 years ago for the Gram-positive bacterium *G. stearothermophilus* where unassembled SLPs were localized in the peptidoglycan (119). This SLP pool in *C. crescentus* appears poised to rapidly repair lattice defects arising from environmental damage, growth, or membrane curvature changes. Interestingly, the study challenges the prevailing view hat LPSs in Gram-negative *C. crescentus* are precoated with the SLP RsaA. Instead, it suggests that unassembled RsaA likely self-integrates into the S-layer lattice after crossing the cell envelope, assembling in the Ca^2+^-rich extracellular milieu. This finding underscores the remarkable capacity of some bacteria to maintain a continuous, defect-minimized S-layer lattice across their entire surface, as also emphasized by von Kügelgen *et al.* (27).

Supporting evidence comes from Oatley *et al.* who investigated S-layer formation in the Gram-positive anaerobe *C. difficile*, although at lower resolution (120). The group overcame significant technical challenges inherent to microscopy under anaerobic conditions. For live imaging, cells were confined beneath a sealed glass-bottom Petri dish to create an oxygen-free environment or fixed at specific time intervals. Using the fluorescent D-amino acid HCC-amino-D-alanine, incorporation of the SLP was tracked with widefield microscopy. Additionally, the team inducibly expressed an immunologically distinct version of SlpA for visualization by immunofluorescence. Their findings revealed that in *C. difficile*, the integration of SlpA is tightly coordinated with peptidoglycan synthesis and restricted to a single cell pole—contrasting with the bipolar insertion observed in *C. crescentus* (118) and *C. glutamicum* (19). Together, these studies highlight the diversity of S-layer assembly strategies across bacterial species and the close coupling between SLP dynamics, cell growth, and morphology.

## Computational workflows for S-layer protein structure prediction

Structural biology is experiencing a transformative era, driven by unprecedented advancements in computational protein structure determination. Since the introduction of neural network-based, template-free structure prediction in 2018, the field has accelerated at a remarkable pace, reaching a pinnacle in 2020 when DeepMind and EMBL-EBI unveiled two groundbreaking innovations: AlphaFold 2 and the AlphaFold Database (AlphaFold DB).

AlphaFold 2 (121, 122), an enhanced iteration of the original AlphaFold 1 (123), has become one of the most prominent convolutional neural networks for protein structure prediction. Complementing this, AlphaFold DB (122, 124) provided the scientific community with an open-access repository of highly accurate predicted structures. Initially containing 360,000 structures from 21 model organisms, AlphaFold DB has since expanded to over 214 million entries (as of September 2024)—vastly outpacing the ∼225,000 experimentally determined structures currently available in the PDB. Moreover, many PDB entries represent only fragments of proteins, underscoring AlphaFold DB’s unprecedented contribution to the field.

While these numbers alone underscore the revolutionary impact of AlphaFold, its predictive accuracy is equally transformative. AlphaFold 2 achieves near-experimental precision, eliminating the need for template-based modeling. At CASP14 (Critical Assessment of Techniques for Protein Structure Prediction)—a reoccurring community-wide competition to assess the performance of structure prediction tools) in 2020 (125), AlphaFold 2 outperformed all competitors with a Global Distance Test Total Score exceeding 90%, a benchmark comparable to experimental precision (126). This marked a 30% improvement over AlphaFold 1’s performance at CASP13 in 2018. Global Distance Test Total Score quantifies similarity between predicted and reference structure models based on alpha carbon distances, with higher scores indicating greater accuracy. Trailing AlphaFold 2 at CASP14 was an updated version of trRosetta (127, 128) from the Baker group, which surpassed AlphaFold 1’s 2018 performance—a testament to the rapid evolution of computational methods in structural biology.

Building on AlphaFold’s foundation, DeepMind has extended its capabilities to predict protein complexes with AlphaFold-Multimer (129) and posttranslationally modified proteins, including glycosylated structures with AlphaFold 3 (38). Concurrently, the Baker group has advanced the field with the recent release of RoseTTAFold All-Atom (130), a neural network that incorporates not only protein sequence data but also ligands and covalent modifications to model complex interactions with unprecedented precision.

Despite their strengths, neural network-based approaches have limitations. For instance, at CASP15 in 2022, classical docking and homology modeling outperformed neural networks in predicting protein–RNA and protein–ligand complexes, highlighting an area ripe for improvement (131). This sets the stage for RoseTTAFold All-Atom and other innovative neural networks to potentially dominate in these categories at CASP16 in December 2024.

Open-source computational tools for SLP structure prediction and evaluation are summarized in Table 2, listed according to their appearance in this review.

**Table 2:** Open-source computational tools applicable to the study of S-layer protein structures

Name	Description	Online source code	Reference
TomTwin	Assisted particle picking for cryo-ET	github.com/MPI-Dortmund/tomotwin-cryoet	(165)
DeepFinder	Assisted particle picking for cryo-ET	github.com/deep-finder	(166)
DeePiCt	Assisted particle picking for cryo-ET, especially suitable for low abundance proteins	github.com/ZauggGroup/DeePiCt	(70)
tomoDRGN	Assisted particle picking for cryo-ET, especially suitable for various structural protein conformations	github.com/bpowell122/tomodrgn	(117)
Alphafold2, Alphafold Multimer, Alphafold3	Protein monomer, multimer, and protein complex structure prediction based on primary sequence	github.com/google-deepmind/alphafold	(38, 121, 122, 129)
RoseTTAFold All-Atom	Protein and protein complex structure prediction based on primary sequence	github.com/baker-laboratory/RoseTTAFold-All-Atom	(130)
SymProFold	Prediction of symmetric assemblies based on the Alphafold pipeline	github.com/symprofold	(31)
AlphaPulldown	Python package for high throughput screening of protein–protein interactions	github.com/KosinskiLab/AlphaPulldown	(145)
AlphaFastPPi	High throughput screening of protein–protein interactions at low computational cost	github.com/MIDIfactory/AlphaFastPPi	(146)
PeSTo/PeSTo-Carbs	Prediction of protein interaction sites	github.com/LBM-EPFL/PeSTo; github.com/LBM-EPFL/PeSTo-Carbs	(147, 148)
RoseTTAFold2-Lite	Large-scale protein–protein interaction prediction	github.com/SNU-CSSB/RF2-Lite	(149)
ColabDock	Protein docking based on structure prediction and experimental data	github.com/JeffSHF/ColabDock	(150, 151)
DeepRank, DOVE, InterPepRank	Scoring of generated protein structures and complexes	github.com/DeepRank/deeprank github.com/kiharalab/DOVE wallnerlab.org/InterPepRank	(98, 152, 153)
DeepMSA2	Generation of custom MSAs for protein structure prediction	zhanggroup.org/DeepMSA	(160)
GlycoSHIELD	Modeling of glycosylated protein structures	github.com/GlycoSHIELD-MD	(161)

### Open-source structure prediction workflows

With great power comes great responsibility—or in this context, groundbreaking advancements must be shared openly to empower the scientific community, fostering collaboration and driving progress.

Encouragingly, the spirit of open collaboration remains strong within in the structural biology community. Recent advances have carved out a dedicated niche for S-layer biologists in the realm of neural network-based structure prediction, providing tools tailored to unique challenges of SLP structure determination. Two notable developments stand out—both released by distinguished teams of experts and poised to revolutionize this subfield. The first, from Johnston and Doye-experts in computational modeling-working with Bharat and Isbilir, leaders in cryo-EM and cryo-ET fields, introduces a specialized computational workflow for SLPs (63). Their approach seamlessly integrates advanced neural networks with experimental structural data, delivering an unparalleled level of precision and adaptability for S-layer structure prediction. The second comes from Pavkov-Keller’s team, experts in S-layer biology, who have leveraged decades of expertise in S-layer structures, to address key challenges specific to S-layer structures (31). These approaches not only streamline structure determination, but the large, high-quality datasets generated by them also enable the exploration of functional dynamics and interactions within these crystalline lattices, linking structural features to biological function.

### Advances and challenges in protein complex structure modeling

Previous efforts to predict the structures of large protein complexes have been hindered by a key limitation—their dependance on *a priori* information, which is often unavailable to researchers studying SLPs.

Among these efforts, Bryant *et al.* (129) and Gao *et al.* (132) developed methods inspired by AlphaFold Multimer, building on the foundation of AlphaFold 2 (133). Bryant *et al.* focused on improving the speed and efficiency of the multiple sequence alignment (MSA) step (129), a key component of AlphaFold 2, while Gap *et al.* bypassed the use of MSAs altogether (132). However, these methods rely heavily on *a priori* knowledge of complex stoichiometry (*i.e.*, number of subunits) and perform poorly with posttranslationally modified proteins, such as cleaved variants—limitations shared with AlphaFold Multimer.

Some AlphaFold 2 integrations take advantage of the similarity between protein–protein interactions and the forces driving protein folding, enabling AlphaFold 2 to predict multimeric protein assemblies. CombFold (134), for example, constructs multimeric complexes hierarchically by combining pairwise subunit interactions predicted by AlphaFold Multimer, integrating experimental distance restraints like cross-linking mass spectrometry or FRET data. Similarly, OpenFold, a PyTorch-based reimplementation of AlphaFold 2, supports training and fine-tuning (135) and is freely accessible on GitHub (github.com/aqlaboratory/openfold).

Uni-Fold Symmetry represents an innovative advance (136) by leveraging the intrinsic symmetry often observed in multimeric complexes—estimated to be present in 72% of the PDB structures (137). This tool models only the asymmetric unit of the complex, using a prespecified symmetry group to generate the full structure, thereby reducing computational demands and offering a more efficient workflow. It is the only open-source platform supporting AlphaFold Multimer training (available at github.com/dptech-corp/Uni-Fold).

Recently, Schweke *et al.* (97) introduced a pipeline that combines AlphaFold 2 with AnAnaS (Analytical Analyzer of Symmetries), a mathematical tool for evaluating symmetry axes (138). This approach enables systematic prediction of homo-oligomeric complexes with cyclic symmetries on a proteome-scale, validated by experimental cryo-EM data (139), highlighting the importance of corroborating computational predictions with experimental data.

Amidst these developments, two novel pipelines developed by Johnston *et al.* (63) and by Buhlheller *et al.* (‘SymProFold’ (31)) stand out for their pioneering focus on SLPs. Unlike previous methods, these pipelines do not rely on *a priori* symmetry or stoichiometry information. Specifically tailored for SLPs, they utilize the unique symmetry and planar restraints characteristic of these protein assemblies, offering promising solutions to long-standing challenges in SLP structure prediction and advancing our understanding of these complex protein lattices.

### SLP-tailored *in silico* prediction workflows

Both the team around the software “SymProFold” and Johnston *et al.* base their work on the Alphafold Multimer pipeline (129) to construct a stable, symmetric unit cell, which they propagate to generate the complete S-layer lattice. Extending their approach, both teams propose adaptations for modeling viral capsids—structures that, like S-layers, consist of repeating planar faces arranged around a curved surface, forming a regular polyhedron. This structural similarity allows the principles of S-layer organization to be applied directly to viral capsids. Central to both methodologies is the planar symmetry intrinsic to SLP oligomers. The workflow depicted in Figure 4 illustrates the streamlined approach for S-layer structure prediction adopted by both teams.

While AlphaFold Multimer excels in predicting dimeric complexes, it struggles with larger multimers, especially those with heteromeric composition (140). This limitation poses a significant hurdle for predicting S-layer lattices composed of two or more distinct SLPs. Relying solely on AlphaFold Multimer for such complex assemblies remains risky, as its predictive accuracy declines with increasing complexity and heterogeneity. Both workflows undergo rigorous validation by comparing predicted models with experimental data, ensuring accuracy before tackling more complex, unexplored structures. Johnston *et al.* (63), for instance, take a progressive approach starting with the modeling of well-characterized SLPs moving to homologs, then exploring poorly defined SLPs, and ultimately uncovering novel SLPs from uncultured microbial lineages. This systematic exploration provides insights into the evolutionary trajectory of prokaryotic S-layers and uncovers overarching themes in their assembly mechanisms. Remarkably, the only required input is the primary amino acid sequence, optionally truncated to the region of interest (*e.g*., by removing signal peptides or domains irrelevant to S-layer assembly), thereby reducing computational complexity. Notably, users are not required to provide prior information on unit cell dimensions or symmetry. However, Buhlheller *et al.* note that predicting p1 lattices without such data remains challenging as simple solutions may be discarded prematurely during the standard workflow.

Both workflows (Fig. 4) treat proteins as individual subchains or as segments split into the N- and C-terminal portions, reflecting the distinct symmetries of the different protein domains. This segmentation not only addresses symmetry-related issues but also reduces computational demands, as predictions scale with chain length. Additionally, full-length intertwined proteins may fail to yield accurate final assembly, since only certain domains contribute to lattice formation, while others play roles in S-layer anchoring—a distinction discussed extensively in this review and previous work (5). For each subchain, potential oligomers, such as homo-dimers, homo-trimers, homo-pentamers, homo-hexamers are predicted, corresponding to p2, p3, p4, and p6 structures, with Johnston *et al.* also considering heptamers and pentamers, which are uncommon but occasionally observed in S-layer lattices due to structural defects (27). At this stage, symmetry-based complexes are ranked and filtered using AlphaFold Multimer’s confidence metrics, particularly the weighted interface predicted Template Modeling (ipTM) + predicted Template Modeling (pTM) score. This metric combines the more heavily weighted ipTM, which evaluates confidence in subunit positioning relative to each other, with the global pTM, which assesses overall model reliability relative to a hypothetical structure. Higher ipTM + pTM scores indicate more accurate predictions (141).

Confidence levels are further visualized using Predicted Alignment Error matrices, which highlight positional uncertainty (in Ångström) for each aligned and predicted residue, providing critical insights into domain packing and relative positioning. High Predicted Alignment Error values pinpoint areas of uncertainty, guiding researchers in assessing domain placement confidence. Models with structural clashes or redundancy (*e.g*., identical models identified through superimposition) are excluded from the pipeline. The final step involves aligning rotational symmetry between the highest-ranking subchain solutions to assemble an optimized primitive unit cell, which is then propagated to construct the complete S-layer lattice. The resulting model is presented as a visually compelling output, accompanied by all relevant parameters, rankings, and supporting data, providing researchers with a robust and interpretable representation of the most probable S-layer architecture.

### Evolutionary insights from structural predictions

The models generated by these two *in silico* pipelines closely match experimentally derived structures from EM, AFM, X-ray crystallography, or cryo-EM.

Buhlheller *et al.* report only a 5% deviation between computationally predicted and reference models across diverse symmetry types, while Johnston *et al.* achieve less than 2% deviation for the *in silico* model of the *C. glutamicum* S-layer compared to a cryo-EM structure. These computational methods also offer molecular insights beyond the resolution limits of imaging techniques like negative staining or freeze-etching EM. For certain S-layers lacking prior structural information—such as those from *Vibrio aerogenes, Paenibacillus naphtalenovorans, Pyrococcus abyssi, Methanococcus voltae, Thermococcus camini, Thermococcus thioreducens,* and *Phocaeicola vulgatus*—the workflows provided provisional high-confidence models (SymProFold (31)). Notably, Buhlheller *et al.* validated two novel S-layer structures, originating from *Viridibacillus arvi* (computationally derived structure shown in Fig. 5*C*) and *M. voltae*, *via* domain-specific X-ray crystallography, while Johnston *et al.* employed cryo-EM and cryo-ET in collaboration with the Bharat group.

The breadth of structures these pipelines can predict (over 150 SLPs in the case of Johnston *et al.*) has enabled key biological insights into structural S-layer biology. Buhlheller *et al.* identified correlations between pore size, barrier function, and lattice flexibility, with larger pores allowing for more lattice flexibility and anchoring segment length providing clues about periplasmic space thickness. Both teams highlight a central theme in S-layer biology: SLPs across species often retain similar conserved domain arrangements, despite lacking sequence or structural homology. For example, several archaeal SLPs share a homodimeric domain arrangement resembling that of *M. acetivorans* (85) (Fig. 5*B*), with previously uncharacterized dimeric anchoring domains revealed by predictive models.

Johnston *et al.* adopt a phylogenetically broad approach, analyzing bacterial and archaeal SLPs to uncover evolutionary patterns and identify novel SLPs in phyla not previously associated with S-layers, such as *Aenigmatarchaeota, Altiarchaeota, Undinarchaeota,* and *Thermodesulfobiota*. These also uncovered novel anchoring mechanisms for bacterial SLPs, resembling those known from non-SLP cell surface proteins, such as putative C-terminal pseudomurein-binding domain in *Methanothermus fervidus* (63). The expansive dataset analyzed by Johnston *et al.* highlights key evolutionary trends in bacterial and archaeal S-layers. In bacteria, S-layers have arisen independently multiple times, resulting in high variability in lattice type, arrangement, and anchoring mechanisms. Symmetries such as p1, p2, p3, p4, and p6 are well-represented, with p6 being most common. Phyla in which most members maintain S-layers, such as *Deinococcota,* are rare, and different symmetries occasionally co-occur within the same phylum, such as *Bacillota*.

By contrast, in archaea, S-layers are more conserved, with consistent structural and anchoring features. Johnston *et al.* categorized archaeal S-layers into three structural classes: hexagonal spiral pyramids, p4 lattices, and hexagonal tile-like configurations. The absence of an S-layer often correlates with alternative protective strategies, such as the double-membrane architecture observed in *Methanobrevibacter, Thermoplasma,* and *Ignicoccus* (63).

These studies collectively reveal that SLPs are more widespread in prokaryotes than previously assumed, particularly in poorly characterized taxonomic lineages. This expanded understanding provides valuable insights into microbial ecology, anchoring mechanisms, interaction interfaces, and pore dimensions. Such information is particularly relevant for pathogenic microorganisms and the development of targeted interventions. For instance, disrupting the S-layer reduces the virulence of *B. anthracis* (28). Furthermore, these pipelines offer a valuable resource for generating *ab initio* S-layer models for unculturable organisms or those reliant on symbiotic growth conditions. Finally, structural and evolutionary insights have broader implications for understanding complex microbial community dynamics, such as biofilms (45, 142).

### Overcoming challenges in modeling hetero-oligomeric complexes

As of this writing, the workflow developed by Johnston *et al.* remains in preprint and is not yet peer-reviewed (63). In contrast, SymProFold (31) is publicly available on GitHub (github.com/symprofold), providing 18 predicted assemblies with detailed annotations, alongside comprehensive documentation, guides, and tutorials. Notably, implementing SymProFold requires the installation of AlphaFold Multimer, either on the same or a compatible computational system.

While both workflows perform well for homomeric S-layer modeling, a significant limitation lies in their inability to predict hetero-oligomeric complexes. This stems from intrinsic constraints within AlphaFold Multimer, which struggles to accurately model S-layers composed of two or more distinct protein species. Such cases are observed in the archaeal genus *Sulfolobus* (11) and the Gram-positives *C. difficile* (29) and *L. acidophilus* (111).

Some organisms require two distinct SLPs for complete lattice formation. For example, the Gram-negative bacteria *T. forsythia* and *Tannerella serpentiformis* rely on TfsA (UniProt A0A1D3UND3) and TfsB (UniProt A0A1D3UN43), and TssA (UniProt A0A2R4KII9) and TssB (UniProt A0A2R4KIF3) (143, 144), respectively, to assemble their complete S-layers. Both proteins likely anchor to the outer membrane *via* LPS, instead of one protein acting as an anchor and presenting the other protein on the surface. This is because both proteins contain a C-terminal structural motif that serves as a targeting signal to the Type IX Secretion System. This motif is cleaved upon export to facilitate lipidation and membrane attachment (143). While p4 lattice symmetry and general unit cell dimensions are known, finer structural details of *Tannerella* spp. S-layers remain unresolved. Such heteromeric systems highlight a major challenge for current computational workflows, emphasizing the need for further refinement to address these complex assemblies.

Buhlheller *et al.* also identify a limitation in the filtering and ranking step of their workflow (31), which prioritizes strong intermolecular interactions to assemble the complete S-layer lattice. As a result, assemblies driven by multiple weaker interactions may remain undetected. Overcoming this bottleneck may require incorporating *a priori* knowledge, such as known interaction sites, stoichiometry, or experimental data. Both Johnston *et al.* and Buhlheller *et al.* acknowledge these imitations and signal promising future updates, which may include improvements for modeling hetero-oligomeric S-layer structures.

### Alphafold multimer integrations applicable to S-layer structural studies

In neural network-based protein–protein interaction prediction, Alphafold Multimer has been incorporated into several pipelines with potential applications in S-layer structure prediction.

One such tool, AlphaPulldown (145), is a versatile Python package for high-throughput screening of protein–protein interactions (available github.com/KosinskiLab/AlphaPulldown). It supports custom structural multimeric templates, protein fragment modeling, and integration of crosslinking mass spectrometry–derived data, with a Jupyter notebook provided for downstream analysis. Similarly, AlphaFastPPi (146) (github.com/MIDIfactory/AlphaFastPPi) enables large-scale proteome-wide interaction screenings across both prokaryotic and eukaryotic species. Optimized for speed, computational efficiency, and reduced environmental impact, it delivers high-quality predictions.

These large-scale tools could aid in identifying interaction partners of SLPs, particularly in organisms where the S-layer is assembled from multiple proteins (*e.g*., *D. radiodurans* (112, 116)) or in cases where dedicated SLPs remain uncharacterized. More broadly, they may uncover novel building blocks of complex cell wall architectures, particularly those involving S-layers as the outermost cell surface layer.

### Neural network-based approaches beyond Alphafold multimer

Other neural network-based approaches for predicting protein–protein interfaces operating independently of Alphafold multimer include PeSTo (available at pesto.epfl.ch) (147), an open-source geometric transformer capable of predicting interaction interfaces between query protein structures and other biomolecules (proteins, nucleic acids, lipids, ions, and small molecules). PeSTo offers fast, high-confidence predictions suitable for high-throughput applications. Its latest extension, PeSTo-Carbs (148), focuses on protein–carbohydrate interactions. However, it is important to note that its true negative rate (the ability to correctly predict the absence of a false interface) is considerably higher than its true positive rate (predicting the presence of a true interface) (0.934 versus 0.713). This is further reflected in the predictive values: the positive predictive value (the proportion of true positives among all positive predictions) is 0.365, while the negative predictive value (the proportion of true negatives among all negative predictions) is 0.984.

RoseTTAFold2-Lite (149) is another large-scale deep learning pipeline designed for rapid, proteome-wide protein–protein interaction and complex structure prediction, leveraging residue–residue coevolution and structural modeling. It has already identified over 250 high-confidence interaction pairs, including several known virulence factors. Similarly, ColabDock (150, 151) is a framework that integrates experimental data into deep learning–based structural predictions without requiring retraining of the neural network.

For scoring *in silico*–generated structures and complexes, that is, identifying the most native structures among the vast array of generated models, DeepRank (98) (available at github.com/DeepRank/deeprank) is an efficient open-source convolutional neural network framework for ranking docking models generated through *in silico* methods and identifying biologically relevant interfaces in crystallographic data, achieving 86% accuracy at the time of publication. Other deep learning scoring strategies include DOVE (152) and graph-based approaches like InterPepRank (153). Physio-chemical methods such as the HADDOCK-mdscore (154, 155, 156, 157) remain valuable complements to deep learning–based scoring frameworks (158).

### Classical docking protocols for SLP complexes

While neural network-based methods have set new standards for protein–protein complex prediction, classical docking approaches leveraging symmetric assembly remain underexplored in SLP modeling. One prominent example is the well-established docking software HADDOCK (154, 155, 156, 157). It supports distance restraints (such as those derived from experimental techniques like NMR or crosslinking) and enforces symmetry constraints, including C1, C2, C3, C4, C6 symmetries, as well as S3 segment triples. A recent addition allows for planarity restraints, penalizing nonplanar assemblies. This software was recently applied to model the attachment of the SlpA S-layer to the *Lactobacillus* cell wall, identifying glycerol-3-phosphate repeats as binding partners based on NMR-derived chemical shift data (111).

### Computational approaches for predicting glycosylated S-layer structures

As discussed, SLPs are often heavily glycosylated, influencing both their tertiary and quaternary structures. AlphaFold 3 (38), the latest iteration of the AlphaFold software, allows for the inclusion of various PTMs, such as phosphorylation, glycosylation, methylation, ion binding (*e.g*., Ca^2+^, Mg^2+^, *etc.*, though Sr^2+^ is not yet supported), and ligand binding (*e.g*., heme, ATP) at specific sites within protein structures or complexes.

However, AlphaFold 3 does not yet support customizable multiple sequence alignments (MSAs), limiting user control over evolutionary information during modeling. Although challenging to generate, custom MSAs can significantly improve model quality in Alphafold 2 (159). Publicly available tools, like DeepMSA2 (160) automate the generation of high-quality MSAs for monomers and multimers through iterative sequence database searches and denoising.

GlycoSHIELD, a recently released open-source toolkit (available at github.com/GlycoSHIELD-MD), enables the modeling of glycosylated proteins (161) by grafting ensembles of *N*- and *O*-glycan conformations onto static protein structures at a lower computational cost than traditional MD simulations. GlycoSHIELD has been validated with proteins such as N-cadherin, the coronavirus spike protein, and GABA receptors and currently supports 68 different glycan types. While it is not suitable for modeling specific glycan–glycan or glycan–protein binding events, it provides coarse glycan “shields” that recover glycan information often lost during cryo-EM data processing. By generating glycan ensembles, this tool accounts for the flexibility and dynamic nature of glycans in biological systems.

Finally, DeepMind has recently unveiled AlphaProteo, a machine learning framework designed for engineering proteins with high-affinity binding to target proteins (162). While AlphaProteo primarily focuses on protein design, its advanced capabilities could also contribute to understanding SLP interactions with their binding partners.

## Summary and future direction

S-layers are self-assembling protein lattices that form the outermost layer of many bacteria and archaea, playing crucial roles in structural integrity, environmental resilience, and pathogenicity. Composed of SLPs, these lattices feature anchoring domains-such as SLH domain and self-assembly domains. Their pore structures adapt to environmental demands, while glycosylation further enhances protective functions and immune evasion, although its full biological role remains incompletely understood.

Recent advances in structural biology, particularly through cryo-EM, cryo-ET, and the integration of computational tools like AlphaFold, have significantly improved our understanding of S-layer architecture and assembly. These methods have yielded high-resolution insights into S-layer structures, revealing the complexity of protein–polysaccharide interactions, calcium ion dependencies, and species-specific attachment mechanisms. Recombinant techniques now enable targeted manipulation of SLPs, and nanobody-assisted strategies have stabilized previously intractable monomers, facilitating their crystallization for structural analysis (Fig. 3).

Notable breakthroughs include the application of AlphaFold Multimer and related computational workflows, which have achieved unprecedented accuracy in modeling S-layer lattices, providing new perspectives on microbial evolution, symmetry, and assembly principles. Nonetheless, challenges persist in predicting large, heterogeneous multimers and PTMs such as glycosylation, areas where emerging computational approaches are beginning to make strides. These techniques, along with the types of samples they are commonly applied to, and the information they deliver, are summarized in Figure 6. Open-source tools for the modeling and analysis of *in silico*–generated SLP structures are presented in Table 2.

To conclude, the integration of advanced imaging, structural, and computational tools is reshaping our understanding of microbial cell surfaces and S-layer biology. These developments not only clarify mechanisms of S-layer assembly, function, and environmental adaptation but also open up exciting avenues for biotechnological applications in synthetic biology and bioengineering. Continued synergy between experimental and computational approaches will deepen our insight into S-layer dynamics and facilitate the design of novel strategies for leveraging these versatile proteinaceous structures in medical and industrial settings.

## Data availability

All data are included within the article.

## Conflict of interest

The authors declare that they have no conflicts of interest with the contents of this article.
